# Supplementary material for: Factors associated with pulmonary tuberculosis in older adults: A scoping review
Source: PLoS One. 2026 Mar 6;21(3):e0344311. doi: 10.1371/journal.pone.0344311 (PMC12965595; doi:10.1371/journal.pone.0344311)
Supplement: Attachment — Submitted filename: Cover_response_PLOS.docx [file pone.0344311.s003.docx]

**Dear Academic Editor**,

We would like to thank the Academic Editor and all reviewers for their careful evaluation of our manuscript entitled *“Factors associated with pulmonary tuberculosis in older adults: a scoping review”*. We are grateful for the constructive and detailed comments, which significantly contributed to improving the clarity, transparency, and methodological rigor of our work.

We have carefully revised the manuscript and provide below a point-by-point response to the main issues raised. All modifications introduced in response to the reviewers’ comments are highlighted in red in the revised manuscript with tracked changes, while the clean version of the manuscript is provided without any markings, as requested.

General response to editorial and reviewer comments

We fully acknowledge the reviewers’ concerns regarding methodological rigor, reporting clarity, cautious interpretation, and alignment with the exploratory nature of a scoping review. In response, we undertook a substantial revision focusing on:

- Improving methodological transparency and internal coherence;
- Refining the description of eligibility criteria, outcomes, and screening procedures;
- Revising inferential and causal language throughout the manuscript;
- Expanding tables and supplementary materials to enhance interpretability;
- Strengthening the limitations and conclusions sections to ensure cautious, evidence-aligned interpretation.

At the same time, we respectfully note that certain core methodological decisions were maintained, as detailed below, to preserve consistency with the study objectives and the evidence mapped.

**1. Search strategy and scope of the review**

We agree that comprehensive and transparent search strategies are essential for scoping reviews. However, after careful consideration, we opted not to modify the original search strategy. The strategy was intentionally designed to balance sensitivity and specificity while aligning with the exploratory objective of mapping factors described in relation to pulmonary tuberculosis in older adults.

Importantly, the search was conducted across multiple international databases and grey literature sources and followed the JBI framework and PRISMA-ScR guidelines. Rather than altering the strategy post hoc, which could introduce methodological inconsistency, we clarified and expanded the description and justification of the search approach in the Methods section to improve transparency and reproducibility.

**2. Inclusion of a prognostic study**

We acknowledge the reviewer’s concern regarding the inclusion of a study with a prognostic outcome. After reassessment, we decided to retain this study, as it provides relevant contextual information on factors described among older adults with pulmonary tuberculosis.

To address this concern, we:

- Explicitly clarified in the Methods and Results that the review includes studies with heterogeneous analytical perspectives;
- Clearly distinguished etiological from prognostic outcomes in the synthesis;
- Revised the narrative to avoid conflation of outcome types.

This approach is consistent with the mapping purpose of a scoping review, which aims to characterize the breadth and nature of available evidence rather than restrict inclusion based on narrowly defined outcome hierarchies.

**3. Methodological heterogeneity and small number of included studies**

We fully agree that the limited number of included studies (n = 6) and their methodological heterogeneity represent important limitations.

Accordingly, we added a dedicated paragraph in the Limitations section explicitly addressing:

- The small number of studies and its implications for robustness and generalizability;
- Variability in study design, definitions of older age, epidemiological settings, and adjustment for confounders;
- Differences in outcome definitions and analytical strategies.

We emphasize that these characteristics reinforce the exploratory nature of the review, and the findings should be interpreted as evidence mapping rather than consolidated or causal inference.

**4. Robustness of data presentation and synthesis**

In response to requests for greater robustness, we expanded the Results section and revised tables to provide clearer information on:

- Exposure and outcome definitions;
- Study design and analytical approaches;
- Contextual and methodological characteristics of included studies.

Although we recognize that scoping reviews do not require formal risk-of-bias assessment, we added a concise discussion of methodological limitations at the study level, as recommended, to support a more nuanced interpretation.

**5. Inter-rater reliability and use of the Kappa coefficient**

We thank the reviewer for highlighting the incorrect description of inter-rater reliability. The manuscript has been revised to correct the terminology and statistical interpretation.

Given that two independent reviewers conducted the screening process, we clarified that Cohen’s kappa coefficient was applied, and we removed any inaccurate references to percentage agreement or Fleiss’ kappa. The revised text now accurately reflects the statistical method used and adheres to standard methodological guidance.

**6. Inferential language and interpretation**

We carefully reviewed the manuscript line by line and replaced inferential or causal language with descriptive, non-inferential terminology consistent with the objectives and design of a scoping review.

At the same time, the core structure and content of the Discussion were intentionally maintained. While we fully agree with the reviewers’ conceptual recommendations, we considered it methodologically appropriate to preserve the Discussion as it is directly grounded in the mapped results. The revisions focused on language refinement and framing, rather than altering the substantive interpretation derived from the evidence presented.

**7. Definition of “older adults”**

We agree with the reviewer that the definition of “older adults” varies across the included studies. In the revised manuscript, we clarified that this scoping review adopted the World Health Organization definition for low- and middle-income countries, which considers individuals aged 60 years or older, a criterion that is also consistent with Brazilian public health and legal frameworks. We further acknowledged that the primary studies operationalized older age heterogeneously, most commonly using thresholds of ≥60 or ≥65 years. Rather than excluding studies based on these differences, we retained all eligible articles in order to comprehensively map how age has been defined and applied in the existing literature. This variability is now explicitly addressed in both the Methods section.

We sincerely appreciate the reviewers’ and editor’s insightful feedback. We believe that the revised manuscript now demonstrates improved methodological coherence, transparency, and interpretative caution, while remaining faithful to the aims and results of the scoping review.

We hope that the revisions adequately address the concerns raised and that the manuscript is now suitable for further consideration by *PLOS ONE*.

Kind regards,

Francisco de Assis Batista, PhD Student
On behalf of all authors
